# Contrasting Roles of Islet Resident Immunoregulatory Macrophages and Dendritic Cells in Experimental Autoimmune Type 1 Diabetes

**DOI:** 10.1371/journal.pone.0150792

**Published:** 2016-03-04

**Authors:** Thomas B. Thornley, Krishna A. Agarwal, Periklis Kyriazis, Lingzhi Ma, Vaja Chipashvili, Jonathan E. Aker, Sarantis Korniotis, Eva Csizmadia, Terry B. Strom, Maria Koulmanda

**Affiliations:** 1 Department of Medicine, Harvard Medical School and the Transplant Institute at Beth Israel Deaconess Medical Center, Boston, Massachusetts, 02215, United States of America; 2 Department of Surgery, Harvard Medical School and the Transplant Institute at Beth Israel Deaconess Medical Center, Boston, Massachusetts, 02215, United States of America; Children's Hospital Boston/Harvard Medical School, UNITED STATES

## Abstract

The innate immune system critically shapes diabetogenic adaptive immunity during type 1 diabetes (T1D) pathogenesis. While the role of tissue-infiltrating monocyte-derived macrophages in T1D is well established, the role of their tissue-resident counterparts remains undefined. We now demonstrate that islet resident macrophages (IRMs) from non-autoimmune mice have an immunoregulatory phenotype and powerfully induce FoxP3+ Tregs in vitro. The immunoregulatory phenotype and function of IRMs is compromised by TLR4 activation in vitro. Moreover, as T1D approaches in NOD mice, the immunoregulatory phenotype of IRMs is diminished as is their relative abundance compared to immunostimulatory DCs. Our findings suggest that maintenance of IRM abundance and their immunoregulatory phenotype may constitute a novel therapeutic strategy to prevent and/or cure T1D.

## Introduction

Autoimmune type 1 diabetes (T1D) arises from T-cell mediated destruction of insulin-producing β-cells. However, alterations in innate immune cells profoundly influence diabetogenic T cell immunity and T1D pathogenesis [1]. In non-obese diabetic (NOD) mice, T1D pathogenesis is associated with elevated inflammatory cytokine secretion by monocyte-derived macrophages [2] and the emergence of an inflammatory Batf3+ intra-islet dendritic cell (DC) subset [3]. In humans, monocytes and myeloid DCs from individuals with T1D have altered TLR responses that give rise to heightened NF-κB activation and pro-inflammatory cytokine secretion [4].

Circulating monocyte-derived, tissue-infiltrating macrophages play a pro-inflammatory role in T1D; however, the role of their tissue-resident counterparts remains undefined. Tissue-resident macrophages (TRMs) are an Ly6C- lineage that arises from yolk-sac derived progenitors [5, 6], distinguishing them from Ly6C+ monocyte-derived macrophages that are replenished by bone marrow-derived circulating progenitors [7]. Recently, we identified a CD169+TIM-4+ TRM subset in heart and skin that migrates upon activation to draining lymph nodes, produces TGFβ, induces FoxP3+ Tregs, and promotes allograft survival [8]. This TRM population joins kindred immunoregulatory Ly6C- subsets of bronchoalveolar TRMs that suppress asthmatic lung inflammation [9] and adipose TRMs that control local inflammation and insulin resistance [10]. Thus, many TRM subsets are immunoregulatory in the steady state; however, the influence of inflammation on the immunoregulatory phenotype and function of TRMs has not been intensively studied.

A CD169+ (MOMA-1+) population has been identified in pre-diabetic NOD mice by immunohistochemistry [11], suggesting that a population of TRMs might be present in the pancreatic islets of T1D-prone mice. However, whether these cells: i) are TRMs, ii) possess immunoregulatory phenotype and function in the steady state, or iii) exhibit phenotypic or functional plasticity, remains untested. To this end, we have identified a CD169+ islet-dwelling TRM subset and analyzed its immunoregulatory phenotype and function in quiescence, inflammation and autoimmune T1D.

## Materials & Methods

### Mice

C57BL/6J (B6), female NOD/LtJ (NOD) and female NOR/LtJ (NOR) mice were obtained from the Jackson Laboratories (Bar Harbor, ME). Founder bm12 (B6(C)-H2-Ab1^bm12^/HkEgJ), B6.g7 (B6.NOD^D17Mit21-D17Mit10^/LtJ), and ABM TCR [12] transgenic mice bearing the FoxP3/GFP reporter mice were maintained in our colony. Founder BDC2.5 TCR transgenic mice on the B6.g7 background [13] were obtained from Drs. Mathis and Benoist (Harvard Medical School, Boston, MA) and maintained as heterozygotes in our colony by paired mating with wild type B6.g7 mice. Glucose tolerance was defined as AUC_100mg/dl_<6,000 and blood glucose <150mg/dl at 120 minutes after i.p. injection of glucose (3g/kg). All mice were certified to be specific pathogen-free conditions. All animal experiments were performed under a protocol reviewed and approved by the Institutional Animal Care and Use Committee at Beth Israel Deaconess Medical Center (Boston, MA) and in accordance with the U.S. Department of Health and Human Services’ Guide for the Care and Use of Laboratory Animals.

### Immunofluorescence microscopy

Pancreases were snap-frozen; stained with rat anti-mouse CD169, goat anti-mouse TIM-4 (R&D Systems, Minneapolis, MN) and a triple antibody cocktail containing rabbit anti-human glucagon, somatostatin, and pancreatic polypeptide. Images were acquired on an LSM 510 Meta using a plan-apochromat 1.3 numerical aperture 40x objective with oil (Carl Zeiss, Thornwood, NY).

### Isolation of islet resident leukocytes

Pancreatic islets were purified as described [14], gravity washed, and incubated at 37°C in HBSS containing 0.25% trypsin, 2mM EDTA, and 0.1mg/ml DNAse with agitation (120 RPM) for 10 minutes. Digestion was stopped by adding FBS to a 20% final concentration and placing samples on ice. Single cell suspensions were then stained with the indicated viability dye and antibodies. Absolute numbers were determined using CountBright absolute counting beads (Life Technologies, Carlsbad, CA).

### FACS analysis and cell sorting

The following monoclonal antibodies were used: anti-CD11b (M1/70), anti-CD11c (N418), anti-CD16/32 (93), anti-CD39 (DMS1), anti-CD45 (30-F11), anti-CD73 (TY/11.8), anti-CD169 (3D6.112), anti-CX3CR1 (SA011F11), anti-F4/80 (BM8), anti-Ly6C (HK1.4), anti-TIM-4 (5G3), anti-CD4 (GK1.5), anti-CD25 (PC61), anti-CD44 (IM7), anti-CD62L (MEL-14), anti-Vα2 (B20.1), anti-Vβ4 (KT4), anti-Vβ8.1/2 (KJ16-133.18), and anti-FoxP3 (FJK-16s). Live/Dead Yellow Fixable Stain, Fixable Viability Dye eFluor 780 or DAPI were utilized to assess viability. Intracellular FoxP3 staining was performed using the Fixation/Permeabilization Staining Buffer Set (eBioscience, San Diego, CA). Data acquisition and/or cell sorting was performed on a special-order 5-laser BD LSRII flow cytometer, Beckman Coulter Gallios, or BD FACS Aria or Aria II instrument. Purity after sorting was routinely >95%. Analysis was performed using FlowJo software. Mean fluorescence intensities (MFIs) were calculated using the geometric mean of the appropriate fluorescence channel in FlowJo. Expansion Indices were determined using the embedded FlowJo algorithm.

### Mixed lymphocyte reactions

Live CD45+Ly6C-CD11c+F4/80+CD16/32+ IRMs, CD45+Ly6C-CD11c+F4/80-CD16/32- IRDCs, or splenic CD11c^hi^CD11b^low^ DCs (typically 2.5 x 10^3^) were FACS sorted and incubated at a 1:20 ratio with FACS-sorted ABM CD4+FoxP3/GFP- T cells or B6.g7/BDC2.5 CD4+CD44-CD25-CD62L^hi^ T cells (typically 0.5 x 10^5^) labeled with CellTrace Violet (Life Technologies). Culture media was IMDM supplemented with 2-mercaptoethanol, 10% heat-inactivated FBS, 20ng/ml IL-2, and penicillin/streptomycin/L-glutamine. The agonistic 1068–56 peptide (KVAPVWVRMME, Genscript, Piscataway, NJ) was added to BDC2.5 cultures at a final concentration of 2.5μM [15]. After 96 h, ABM CD4+Vα2+Vβ8+ or BDC2.5 CD4+Vβ4+ transgenic T cells were stained with the indicated antibodies. LPS from *E*. *coli* was re-purified as previously described [16] and added to cultures at a final concentration of 1μg/ml.

### Quantitation of IL-6 in supernatants

IL-6 was measured in the supernatants of MLR cultures using a cytokine bead array. The concentration of the experimental samples was interpolated using a 4-parameter logistic non-linear regression model.

### Real-time PCR

Cells were FACS-sorted or resuspended into cell lysis buffer. cDNA was synthesized from column-purified RNA and pre-amplified for 10 cycles using the PreAmp (Life Technologies) target-specific pre-amplification system. Real-time PCR was performed using TaqMan probe/primer sets from Life Technologies (Table 1). Data were normalized to GAPDH using the ΔCT method.

**Table 1:** Table of probe/primer sets used for real-time PCR.

Gene	Protein	TaqMan Assay ID
*Csf1r*	CSF1R	Mm01266652
*Entpd1*	CD39	Mm00515447
*Ccl2*	CCL2/MCP-1	Mm00441242
*Il1b*	IL-1β	Mm00434228
*Il6*	IL-6	Mm00446190
*Gapdh*	GAPDH	4352339E

### Statistics

Statistical analyses were performed with Prism software (GraphPad, La Jolla, CA). Means of two groups were compared by an unpaired t test. Means of three or more groups were compared using a one-way ANOVA with Bonferroni’s post-test. All error bars are +/- S.D. Values of p < 0.05 were deemed statistically significant.

## Results

### CD169+TIM-4+ cells are located at the islet perimeter in C57BL/6 and NOD mice

**Fig 1:**
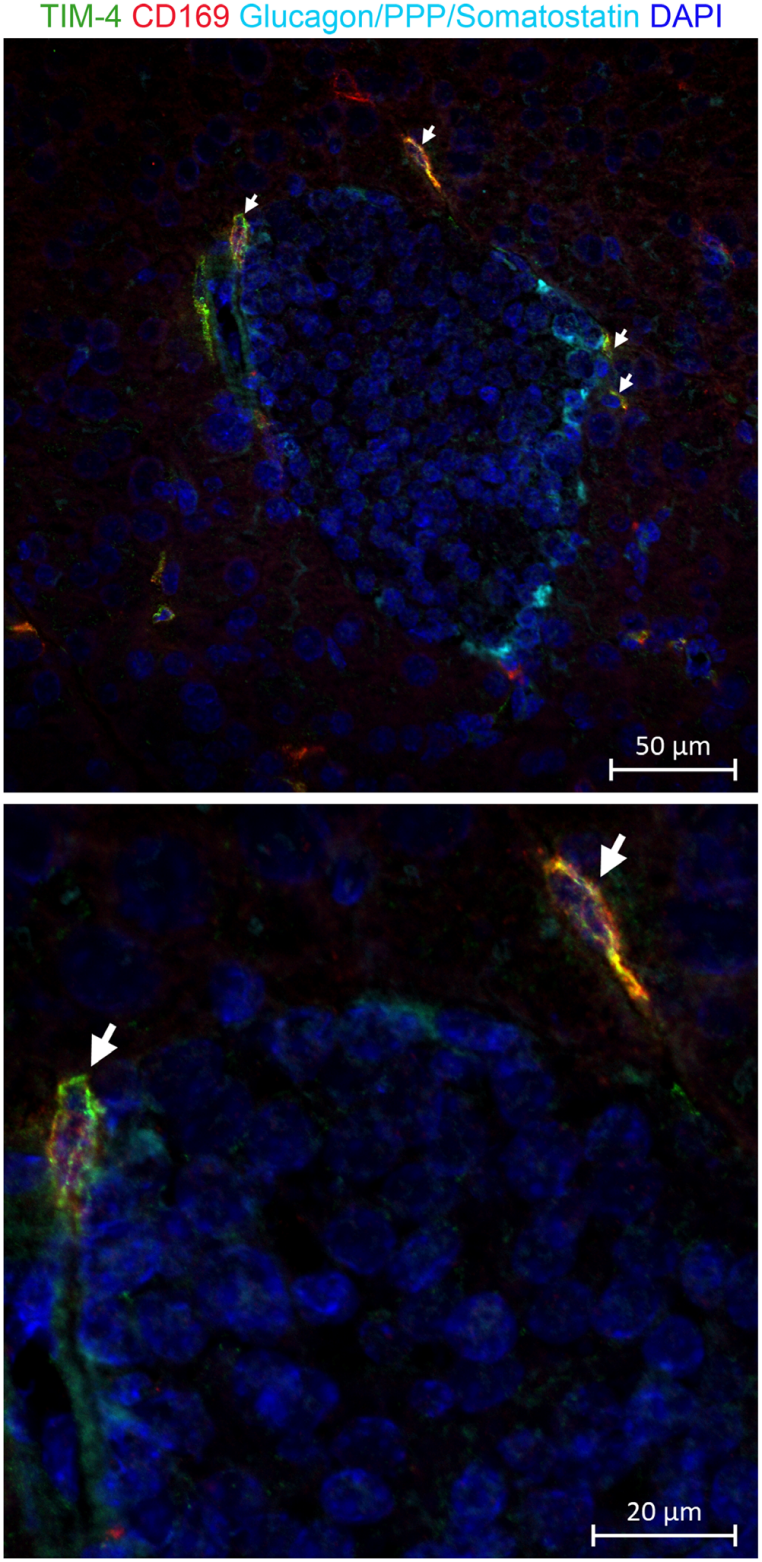
TIM-4+CD169+ cells are present at the islet perimeter in C57BL/6 mice. Frozen pancreas sections from C57BL/6 mice (n = 5) were stained with a triple antibody cocktail reactive to glucagon, somatostatin, and pancreatic polypeptide (Light Blue); anti-TIM-4 (Green); anti-CD169 (Red); and Hoechst (Blue). White arrows indicate CD169+TIM-4+ cells. Individual panels are shown in S1 Fig. Photomicrographs were taken using 40x objective magnification. The bottom panel shows an enlarged area of the photomicrograph in the top panel.

To assess whether a CD169+TIM-4+ TRM population exists in pancreatic islets, we performed immunofluorescence staining on pancreas from C57BL/6 (B6) mice and NOD mice. B6 mice are completely free of insulitis and T1D. NOD mice spontaneously develop invasive insulitis by 5 weeks-of-age and, beginning at 12 weeks of age, develop T1D. We determined the relative position of CD169+TIM-4+ cells with respect to pancreatic islets by co-staining frozen pancreas sections with anti-CD169, anti-TIM-4, and a triple antibody cocktail that stains endocrine cells expressing glucagon (α), somatostatin (δ), and pancreatic polypeptide (γ). We routinely observe CD169+TIM-4+ (Fig 1 and S1 Fig) cells at the islet perimeter in B6 mice. CD169+TIM-4+ cells are increased in abundance but remain at the islet perimeter in pre-diabetic and diabetic NOD mice (S1 Fig).

### Islet resident macrophages are Ly6C-CD11c+CD16/32+F4/80+

**Fig 2:**
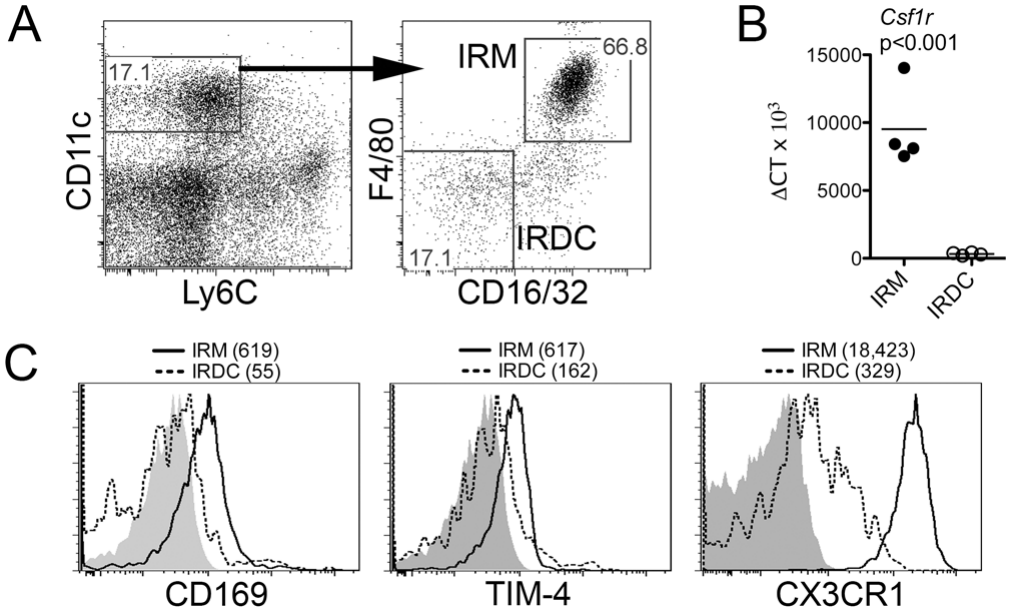
Intra-islet CD45+CD11c+Ly6C-F4/80+CD16/32+ cells from C57BL/6 mice exhibit a tissue-resident macrophage phenotype. Purified C57BL/6 pancreatic islets were enzymatically digested and stained with anti-CD45, anti-CD11c, anti-Ly6C, anti-F4/80, and anti-CD16/32. (A) Identification of live CD45+ (not shown) CD11c+Ly6C- (left panel) cells that exhibit an islet-resident macrophage (IRM, F4/80+CD16/32+) or DC (IRDC, F4/80-CD16/32-) phenotype (right panel) are shown. (B) FACS-sorted IRMs and IRDCs were analyzed for *Csf1r* transcript levels by real-time PCR. Data from independent experiments (n = 4 per group) and their mean are plotted; groups were compared with an unpaired t test. (C) Histograms show protein expression of CD169, TIM-4, and CX3CR1 on gated IRM (solid line) and IRDC (dashed line) subsets. Shaded histograms are the fluorescence minus one (FMO) controls. Geometric mean fluorescence intensities (MFIs) are indicated parenthetically. Data are representative of three independent experiments.

TRM subsets are often identified by a complex extended phenotype that consists of a lack of Ly6C expression [10] as well as the presence of multiple macrophage markers, including CD11c and, F4/80, [9, 17]. While more broadly expressed in mice, CD16 (FcγRIII) and CD32 (FcγRII) are expressed highly on TRM populations in humans [17, 18] and may be useful to identify murine TRMs in conjunction with other markers [19]. To identify and isolate putative TRMs from pancreatic islets, purified B6 and NOD islets were enzymatically digested and analyzed for subsets expressing CD45, Ly6C, CD11c, CD16/32, and F4/80 by flow cytometry. Within the viable CD45+Ly6C-CD11c+ fraction (Fig 2A, left panel), we observe two distinct populations. We designate the dominant Ly6C-CD11c+CD16/32+F4/80+ subset (Fig 2A, right panel) as islet resident macrophages (IRMs). A smaller Ly6C-CD11c+CD16/32-F4/80- subset (Fig 2A, right panel) is designated as islet resident dendritic cells (IRDCs), insofar as these cells are F4/80- and exhibit DC-like functional properties that will be described later. Validating our designation of the extended Ly6C-CD11c+CD16/32+F4/80+ phenotype as a TRM subset, FACS-sorted B6 IRMs express more *Csf1r* transcripts (Fig 2B) and higher levels of the TRM markers CD169, TIM-4 and CX3CR1 (Fig 2C) as compared to IRDCs. NOD IRMs exhibit a similar TRM-like phenotype (S2 Fig).

### Islet resident macrophages powerfully induce FoxP3+ Tregs in vitro but their immunoregulatory function is compromised by TLR4 stimulation

**Fig 3:**
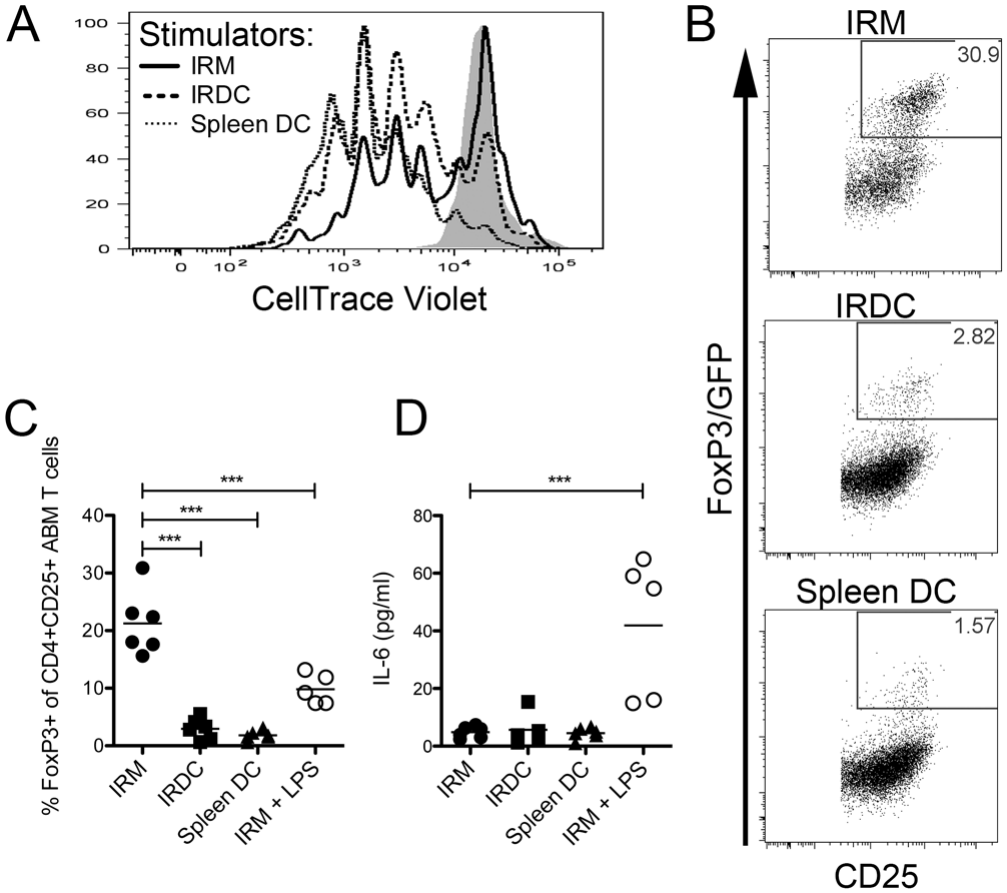
Islet resident macrophages induce Tregs through a mechanism that is dampened by TLR4 stimulation. FACS-sorted ABM CD4+FoxP3/GFP- TCR transgenic T cells were CellTrace violet labeled and cultured with FACS-sorted live IRMs (CD45+Ly6C-CD11c+F4/80+CD16/32+), IRDCs (CD45+Ly6C-CD11c+F4/80-CD16/32-), or splenic DCs (CD11c^hi^CD11b^low^) from bm12 (I-A^bm12^) mice for 96 h. (A) Proliferation of gated live CD25+CD4+Vα2+Vβ8+ ABM TCR transgenic T cells stimulated with IRM (solid line), IRDC (long dashed line), or splenic DC (short dashed line) stimulators is depicted in a histogram. The shaded histogram represents the unstimulated control. Representative data from 3 independent experiments are shown. (B) FoxP3/GFP expression in gated live activated CD25+CD4+Vα2+Vβ8+ ABM TCR transgenic T cells is shown from a representative experiment. (C) FoxP3/GFP expression in gated live activated CD25+CD4+Vα2+Vβ8+ ABM TCR transgenic T cells from independent experiments (n = 4–6 per group) are shown in scatter plots. Groups were compared by ANOVA (p<0.01) and Bonferroni’s post-test (***, p<0.001). (D) Concentration of IL-6 (pg/ml) in the supernatant of independent MLR cultures (n = 4–6 per group) is shown in a scatter plot. Groups were compared by ANOVA (p<0.01) and Bonferroni’s post-test (***, p<0.001).

To test whether IRMs from insulitis and T1D-free mice are functionally immunoregulatory, we performed mixed lymphocyte reactions (MLRs) with IRM, IRDC or splenic DC stimulator cells from bm12 mice (I-A^bm12^) and FoxP3/GFP-CD4+ T cells from ABM TCR transgenic mice. ABM mice express a clonotypic anti-I-A^bm12^ transgenic TCR and a FoxP3/GFP reporter [12]. After 4 days in the MLR, CD4+ ABM T cells stimulated by bm12 IRM, IRDC or splenic DC stimulator cells were collected and analyzed for proliferation by CellTrace Violet dilution and Treg conversion by *de novo* FoxP3/GFP reporter expression. At the 1:20 stimulator:T cell ratio used in our experiments, we observed inter-experimental variability in the frequency of non-activated T cells that failed to upregulate CD25. As proliferation and Treg conversion cannot be determined for this non-activated CD25- population, which likely failed to encounter antigen, we performed proliferation and Treg conversion analyses using an activated (CD25+) CD4+ TCR transgenic T cell gate. Whereas IRMs induce less proliferation as compared to IRDCs or splenic DCs (Fig 3A), IRMs induce a much higher frequency of FoxP3/GFP expression (Fig 3B and 3C; p<0.001). Strikingly, stimulation of the MLR by LPS, a TLR4 agonist, reduces the frequency of FoxP3/GFP+ T cells (20.9% for untreated IRMs vs. 9.3% for LPS-stimulated IRMs, p<0.01; Fig 3C). This decrease in FoxP3+ Treg frequency correlates with an increase in the Treg inhibitory cytokine IL-6 in the supernatant (Fig 3D; p<0.001).

### TLR4 activation compromises the immunoregulatory phenotype of islet resident macrophages

**Fig 4:**
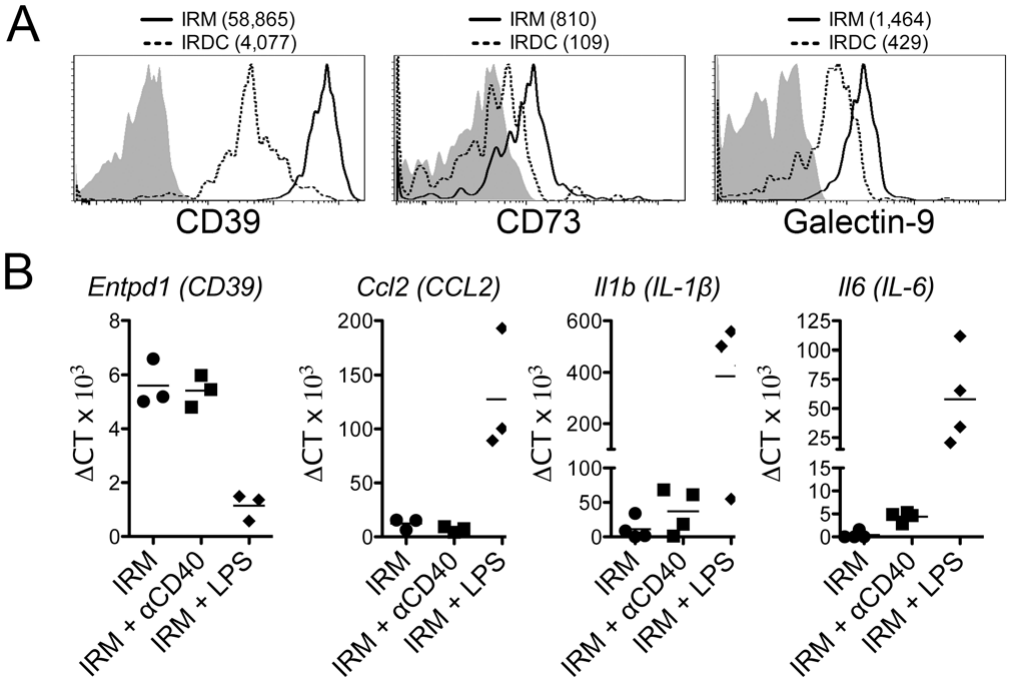
Islet resident macrophages possess an immunoregulatory phenotype that is compromised by TLR4 activation. (A) IRMs (solid line) and IRDCs (dashed line) were analyzed directly *ex vivo* by flow cytometry for CD39, CD73 and galectin-9 using the CD45+Ly6C-CD11c+F4/80+CD16/32+ gate. Shaded histograms are the FMO controls and MFIs are indicated parenthetically. Representative data are shown from one of three independent experiments. (B) FACS-sorted B6 IRMs were stimulated with 1 μg/ml of LPS or 10 μg/ml of agonistic anti-CD40. After 72 h, mRNA from cultured cells was analyzed by real-time PCR for *Entpd1* (CD39), *Ccl2*, *Il1b*, and *Il6* transcripts. Each point represents data from an independent experiment (n = 3–4 per group).

We next determined whether TLR4 activation compromised the immunoregulatory phenotype of IRMs from non-autoimmune B6 mice. At baseline, B6 IRMs relative to IRDCs express more CD39 and CD73, which collectively cleave pro-inflammatory ATP into anti-inflammatory adenosine [20], and galectin-9, which induces apoptosis in TIM-3+ effector T cells [21] (Fig 4A). IRMs stimulated with LPS dramatically downregulate immunoregulatory *Entpd1* (CD39) transcript expression and upregulate pro-inflammatory *Ccl2*, *Il6*, and *Il1b* transcript expression relative to untreated control IRMs (Fig 4B). In contrast to TLR4 stimulation, anti-CD40 stimulated IRMs maintain *Entpd1* transcript expression and only modestly upregulate *Il6* and *Il1b* transcript expression (Fig 4B). An in increase in IL-6 production by LPS stimulated (13.94±4.38pg/ml) as compared to both unstimulated (2.20±0.34 pg/ml, p<0.001 in Bonferroni’s post test) and anti-CD40 stimulated (2.74±0.31pg/ml, p<0.001 in Bonferroni’s post test) IRMs was confirmed by cytometric bead array (ANOVA p<0.001). As IRMs became adherent to the plate during culture, a decrease in CD39 expression could not be validated at the protein level.

### IRMs from euglycemic NOD, NOR and B6.g7 mice robustly induce FoxP3+ Tregs in diabetogenic BDC2.5 T cells

We next tested whether T1D-prone NOD IRMs are inherently defective in their ability to induce FoxP3+ T cells as compared to IRMs from non-T1D strains. To test this, we performed MLRs utilizing FACS-sorted naïve CD4+CD25-CD44-CD62L^hi^ BDC2.5 TCR transgenic responder T cells [13, 22], which express a diabetogenic (I-A^g7^) clonotypic TCR [15], and peptide-pulsed FACS-sorted IRMs or splenic DCs from B6.g7 (H-2^g7^), NOR (H-2^g7^), NOD (H-2^g7^) or B6 (H-2^b^) mice. B6.g7 mice contain the NOD MHC region (H-2^g7^) but, like B6 mice, are insulitis and T1D-free [23]. Congenic NOR mice contain B6 introgressions on chromosomes 2, 4, 11, and 12, exhibit non-invasive peri-islet insulitis, and do not develop T1D [24]. As noted above, FoxP3 conversion was assessed using an activated (CD25+) CD4+ TCR transgenic T cell gate in order to eliminate the confounding influence of the non-activated CD25- T cells fraction.

**Fig 5:**
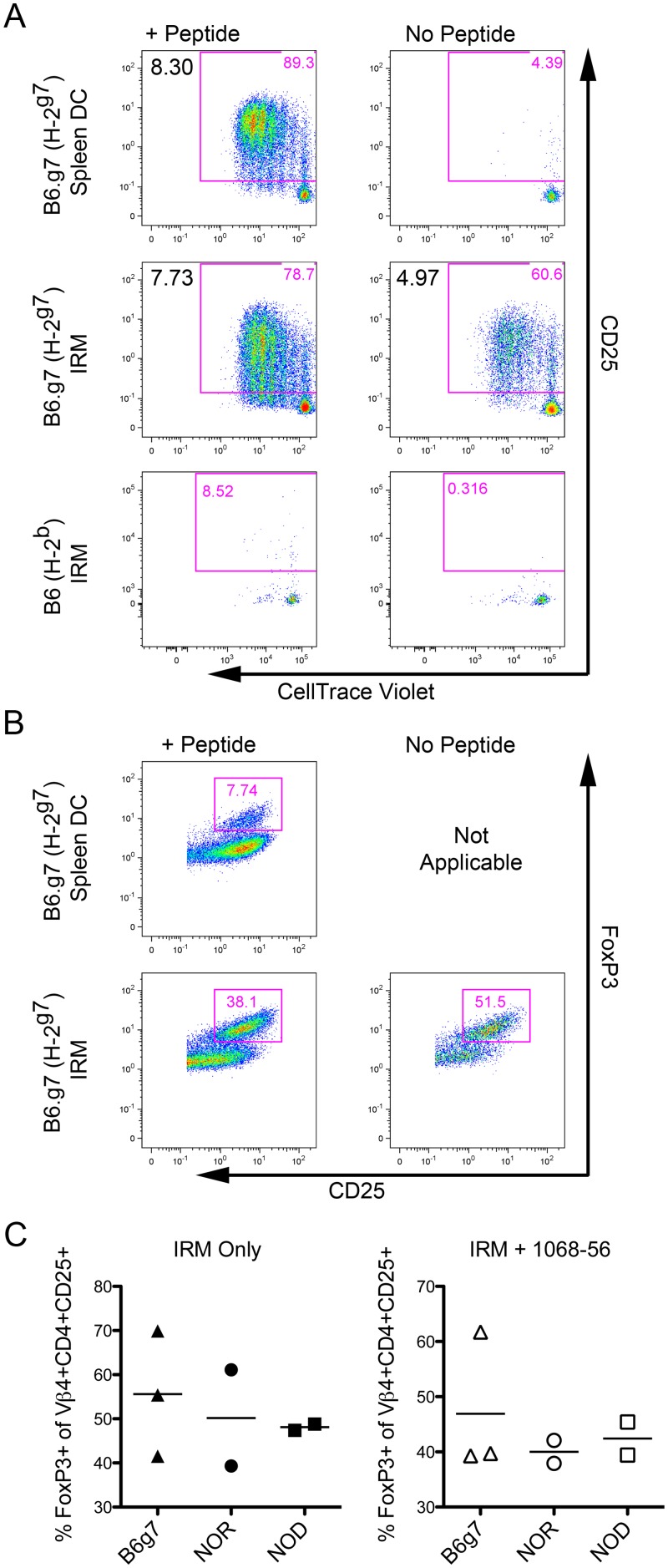
IRMs from B6.g7, NOD, and NOR induce FoxP3+ Tregs in the diabetogenic BDC2.5 TCR transgenic clone. CellTrace Violet labeled CD4+CD25-CD44-CD62L^hi^ naïve T cells from B6.g7/BDC2.5 TCR transgenic mice were cultured for 96 h with IRMs or CD11c^hi^CD11b^low^ splenic DCs from the indicated strain pulsed with 2.5μM of the indicated agonist peptide. (A) CD25 upregulation and proliferation are shown for gated live CD4+Vβ4+ transgenic BDC2.5 T cells stimulated with B6.g7 (H-2^g7^) splenic DCs, B6.g7 (H-2^g7^) IRMs, or B6 (H-2^b^) IRMs pulsed with the agonistic 1068–56 peptide (left column) or no peptide (right column). The expansion index for the gated live activated CD4+Vβ4+CD25+ population was determined using FlowJo analysis software and is noted in the upper-left corner. (B) FoxP3 expression is shown for the gated live activated CD4+Vβ4+CD25+ population. (C) Percent of gated live CD4+Vβ4+CD25+ transgenic T cells that acquire FoxP3 expression after culture with IRMs from the indicated strain are shown from independent experiments (n = 3–4 per strain). A One Way ANOVA indicated no significant difference among groups with (p = 0.95) or without peptide (p = 0.64).

Unpulsed IRMs from mice bearing the H-2^g7^ MHC region induce activation, proliferation (Fig 5A), and robust FoxP3 expression (Fig 5B) in naïve BDC2.5 T cells. Proliferation is increased in peptide-pulsed IRMs as compared to unpulsed IRMs. Moreover, while peptide-pulsed IRMs induce a lower frequency of FoxP3+ Tregs relative to unpulsed IRMs, both unpulsed and peptide-pulsed IRMs induce much more abundant FoxP3 expression compared to peptide-pulsed splenic DCs. Interestingly, robust FoxP3 expression is induced in peptide-pulsed and unpulsed by IRMs from euglycemic NOD IRMs as well as NOR and B6.g7 control IRMs (Fig 5C).

### IRMs from T1D NOD mice undergo phenotypic plasticity and express less immunoregulatory CD39 as compared to IRMs from NOR mice

**Fig 6:**
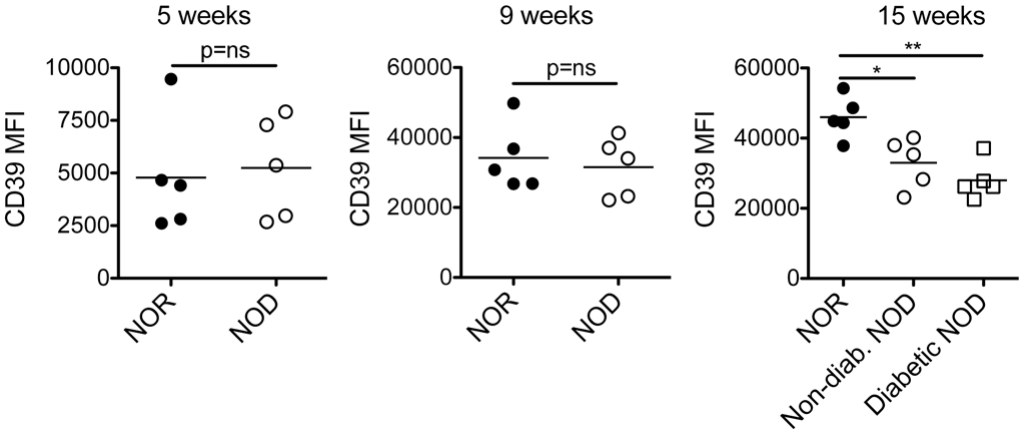
Decreased CD39 protein expression by islet resident macrophages heralds T1D in NOD mice. The MFI of CD39 expression on IRMs from NOD and NOR mice (n = 5 per group pooled from 2 independent experiments) was analyzed by flow cytometry using the CD45+Ly6C-CD11c+F4/80+CD16/32+ gate. Since each time-point was acquired separately, inter-graph comparison of MFIs is not possible. NOD groups were compared to the NOR control group using an unpaired t test (*, p<0.05, **, p<0.01).

We next tested the hypothesis that IRMs, a cell population whose phenotype is altered by TLR4 stimulation, might manifest changes in phenotype associated with age that herald T1D onset. Interestingly, expression of CD39 is reduced on IRMs in diabetic (p<0.01) and non-diabetic NOD (p<0.05) mice as compared to control NOR mice at 15 weeks of age (Fig 6). In contrast, expression of CD39 is similar between NOD and NOR mice at earlier time-points (Fig 6, p = N.S.).

### The balance of islet resident APCs tilts from islet resident macrophages to islet resident DCs in T1D NOD relative to control mice

We next tested the hypothesis that the balance of intra-islet IRMs to DCs is altered during T1D pathogenesis. To test this, we enumerated intra-islet APC populations in 5-, 9-, and 15-week-old NOD mice and age-matched NOR control mice. NOD mice exhibit mild peri-islet insulitis at 5 weeks, moderate peri-islet insulitis at 9 weeks, and invasive insulitis at 15 weeks. Fifteen-week-old NOD mice were stratified into non-diabetic, diabetic, and glucose intolerant cohorts based on the results of an i.p. glucose tolerance test (IPGTT) one day before analysis.

**Fig 7:**
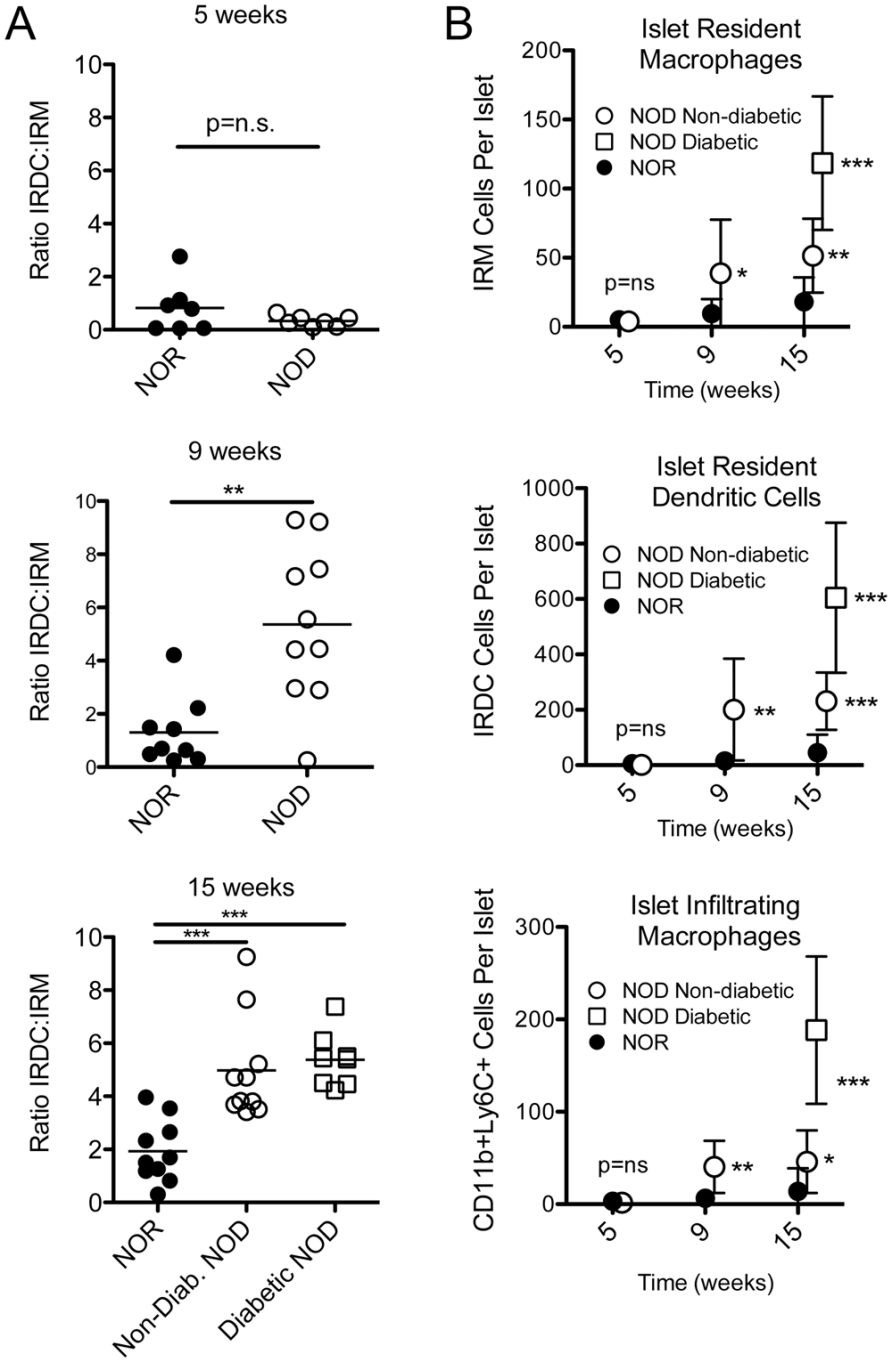
A decrease in the proportion of immunoregulatory islet resident macrophages relative to immunostimulatory islet resident DCs heralds T1D in NOD mice. (A) The ratio of immunoinflammatory IRDCs to immunoregulatory IRMs was calculated at the indicated time-points. Data from individual mice (n = 7–10 per group) were pooled from 3–4 independent experiments. Data were compared with an unpaired t test (**, p<0.01; ***, p<0.001). (B) The absolute number of IRMs (CD45+Ly6C-CD11c+F4/80+CD16/32+), IRDCs (CD45+Ly6C-CD11c+F4/80-CD16/32-), and islet-infiltrating macrophages (CD45+Ly6C+CD11b+) were calculated per islet for NOD and NOR control mice at the indicated ages. Means +/- S.D. are shown for the same cohorts displayed in panel A. NOD groups were compared to the NOR control group utilizing an unpaired t test (*, p<0.05; **, p<0.01; ***, p<0.001).

The ratio of immunoinflammatory IRDCs to IRMs is virtually identical in young 5-week-old NOD and NOR mice (Fig 7A, p = N.S.). However, the ratio of IRDCs to IRMs is dramatically increased in 9-week-old (p<0.01) and 15-week-old (p<0.001) NOD mice relative to age-matched NOR mice. In order to determine whether the increased frequency of IRDCs (CD45+Ly6C-CD11c+CD16/32-F4/80-) relative to IRMs (CD45+Ly6C-CD11c+CD16/32+F4/80+) is due to a decrease in IRMs and/or an increase in IRDCs, we quantified the absolute number of IRMs and IRDCs per islet in each group. At 9 and 15 weeks of age, IRMs, IRDCs, and tissue-infiltrating (CD11b+Ly6C^hi^) macrophages are all increased in absolute abundance in NOD as compared to NOR mice (Fig 7B). In contrast, all three populations are indistinguishable in absolute number between young 5-week-old NOD and NOR mice (Fig 7B).

## Discussion

Immunoregulatory TRM subsets are present in skin, heart [8], lung [9], and adipose tissue [10]. We now report the characterization of an IRM population with immunoregulatory phenotype and function. As T1D approaches, the immunoregulatory phenotype of IRMs is diminished as is their relative abundance as compared to immunoinflammatory DCs. Both of these changes are likely relevant to the onset of overt T1D.

Relative to IRDCs, IRMs more abundantly express immunoregulatory CD39, CD73, and galectin-9. The role of other T cell checkpoints, including PD1/PDL1 [25] and ICOSL [26], in IRM biology is not certain and warrants further study as these pathways play an important role in regulating autoimmune T1D pathogenesis. Compared to DCs, IRMs also more powerfully induce FoxP3+ Tregs in the MLR. However, TLR4 activation reduces FoxP3+ Treg induction by IRMs, possibly related to the downregulation of immunoregulatory CD39 transcripts and the upregulation of pro-inflammatory CCL2, IL-6, and IL-1β transcripts. Corroborating our in vitro findings, there is an age-related loss of CD39 expression by IRMs in 15-week-old NOD mice relative to diabetes-resistant controls. However, IRMs from NOD, NOR, and B6.g7 mice are similar in their ability to induce FoxP3+ Tregs in vitro, suggesting that IRMs from NOD mice are not inherently defective in their ability to induce Tregs.

It is still possible that IRM plasticity compromises other immunoregulatory functions such as attenuating local inflammation [9, 10]. Loss of CD39 function, and the catabolism of pro-inflammatory ATP into anti-inflammatory adenosine as a consequence, is associated with other T cell dependent autoimmune diseases [27]. TRMs from type 2 diabetic mice undergo pro-inflammatory phenotypic plasticity [28]. Thus, IRM plasticity provides new potential mechanisms by which TLR activation may incite T1D [29, 30] and warrants closer examination.

The absolute number of IRMs increases but its proportion relative to inflammatory IRDCs is diminished in non-diabetic and diabetic NOD mice. This suggests that a shift in the balance of intra-islet APCs from one that favors IRMs to one that favors inflammatory IRDCs heralds T1D. As the NOD and NOR differ at defined loci on chromosomes 2, 4 11, and 12, it seems probable that one or more of these genetic intervals drives changes in the balance of intra-islet APCs and, as a result, disease outcome.

The relationships and interactions between IRMs and other immunoregulatory lymphocyte populations, such as regulatory B cells (Bregs), remains unclear. Like Tregs, Bregs play an important role in protection from T1D [31–33]. While TRMs are known to recruit anti-inflammatory resident B1 cells to the peritoneum [34] and to induce germinal center B cell responses in the spleen [35], the impact of TRMs on immunoregulatory B cell development remains uncertain and warrants further study.

We now demonstrate the existence of a functionally immunoregulatory population of islet resident macrophages in non-autoimmune and T1D NOD mice. As T1D approaches, the immunoregulatory phenotype of IRMs is diminished as is their relative abundance as compared to immunoinflammatory DCs. These results suggest that the maintenance of IRM abundance and their immunoregulatory phenotype constitutes a potential therapeutic strategy to prevent and/or cure T1D.

## Supporting Information

S1 Fig TIM-4+ and CD169+ cells are located at the islet perimeter in pre-diabetic NOD mice. Frozen pancreas sections from B6, non-diabetic NOD and diabetic NOD mice (n = 4–5 per strain) were stained with a triple antibody cocktail reactive to glucagon, somatostatin, and pancreatic polypeptide (Light Blue); anti-TIM-4 (Green); anti-CD169 (Red); and DAPI (Blue). White arrows indicate CD169+TIM-4+ cells. Photomicrographs were taken at 40x objective magnification.
(TIF)

S2 Fig Intra-islet CD45+CD11c+Ly6C-F4/80+CD16/32+ cells exhibit a tissue-resident macrophage phenotype in NOD mice. Histograms showing the expression of the tissue-resident macrophage markers CD169, TIM-4, and CX3CR1 on IRMs (solid line) isolated from non-diabetic NOD mice. Shaded histograms are the FMO controls. Data are representative of two independent experiments.
(TIF)

## Abbreviations

DC: Dendritic Cell
FMO: Fluorescence Minus One
IRDC: Islet Resident Dendritic Cell
IRM: Islet Resident Macrophage
MFI: Mean Fluorescence Intensity
NA: Numerical Aperture
NOD: Non-obese Diabetic
NOR: Non-obese Resistant
T1D: Type 1 Diabetes
Treg: Regulatory T cell
TRM: Tissue-Resident Macrophage
