# Supplementary figures and images for: Contrasting Roles of Islet Resident Immunoregulatory Macrophages and Dendritic Cells in Experimental Autoimmune Type 1 Diabetes
Source: PLoS One. 2016 Mar 4;11(3):e0150792. doi: 10.1371/journal.pone.0150792 (PMC4778921; doi:10.1371/journal.pone.0150792)

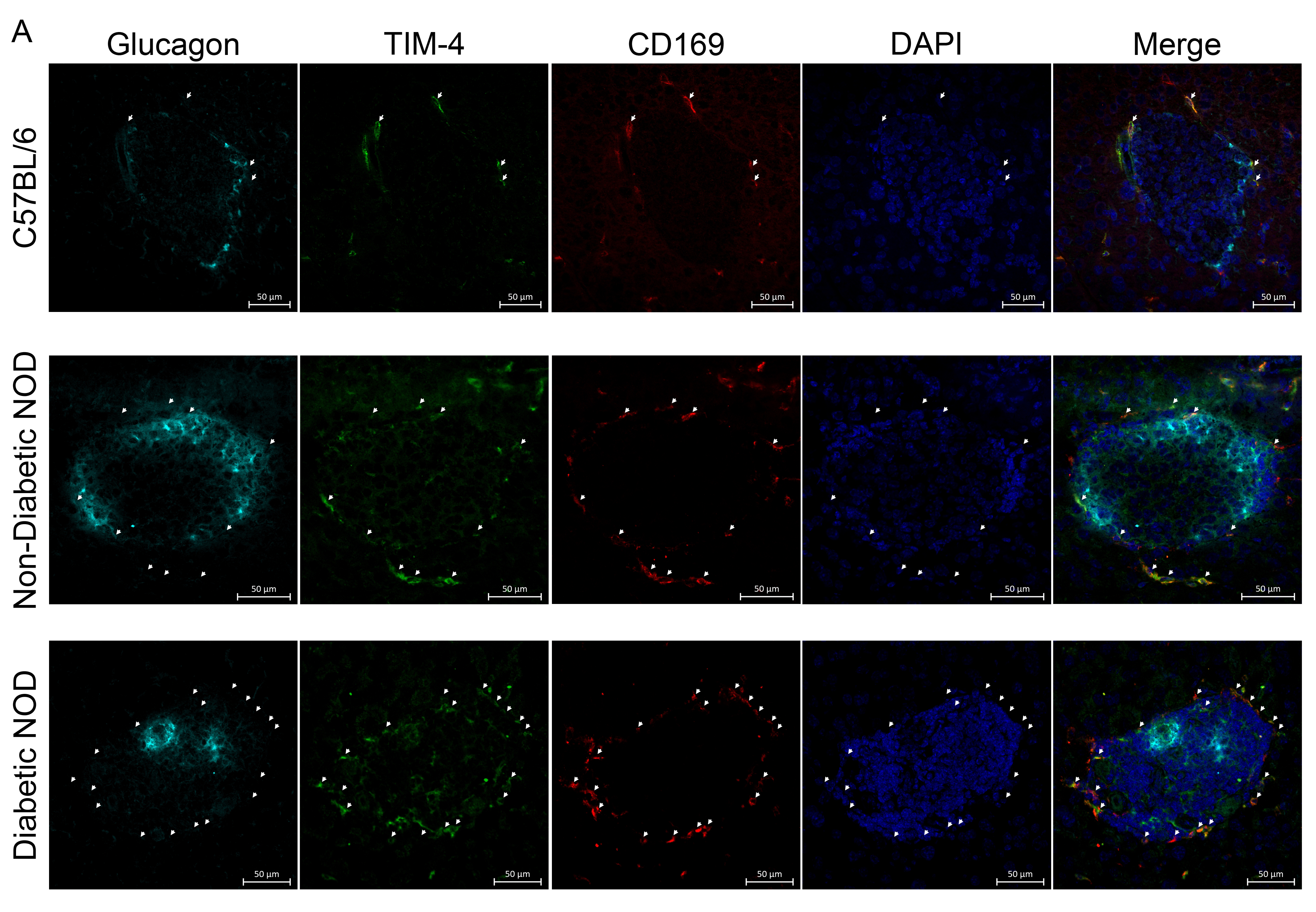

Supplement: S1 Fig — Frozen pancreas sections from B6, non-diabetic NOD and diabetic NOD mice (n = 4–5 per strain) were stained with a triple antibody cocktail reactive to glucagon, somatostatin, and pancreatic polypeptide (Light Blue); anti-TIM-4 (Green); anti-CD169 (Red); and DAPI (Blue). White arrows indicate CD169+TIM-4+ cells. Photomicrographs were taken at 40x objective magnification. (TIF) [file pone.0150792.s001.tif]

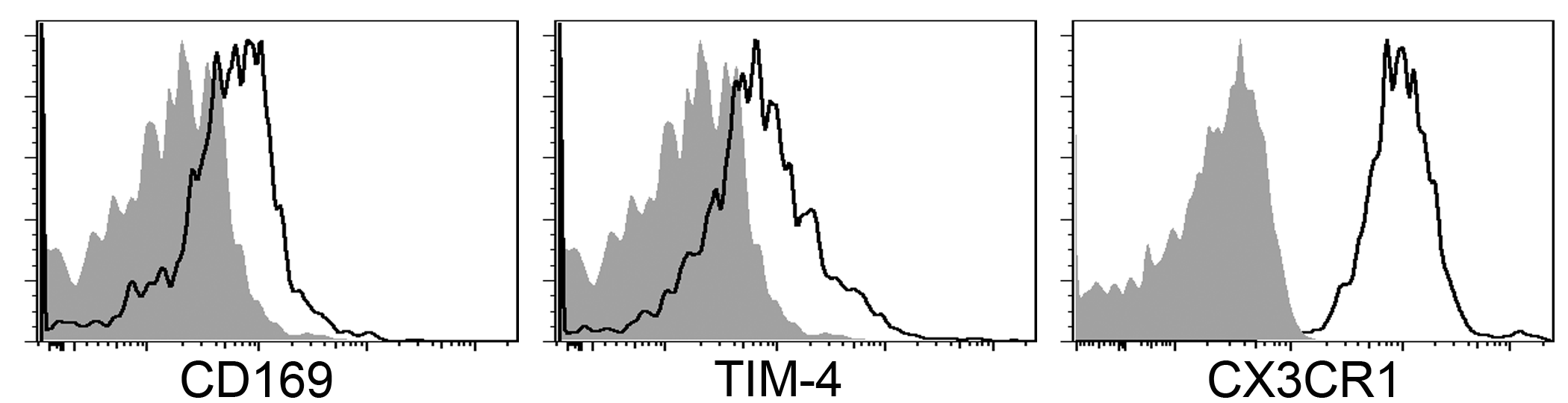

Supplement: S2 Fig — Histograms showing the expression of the tissue-resident macrophage markers CD169, TIM-4, and CX3CR1 on IRMs (solid line) isolated from non-diabetic NOD mice. Shaded histograms are the FMO controls. Data are representative of two independent experiments. (TIF) [file pone.0150792.s002.tif]
